# Supplementary material for: MetaRibo-Seq measures translation in microbiomes
Source: Nat Commun. 2020 Jun 29;11:3268. doi: 10.1038/s41467-020-17081-z (PMC7324362; doi:10.1038/s41467-020-17081-z)
Supplement: Supplementary file 10 — Supplementary Data 7 [file 41467_2020_17081_MOESM10_ESM.zip › File2/Confidence_High_Taxonomy/378054_out.krona.html]

Javascript must be enabled to view this page.

members
magnitude
magnitudeUnassigned
count
unassigned
taxon
rank

378054\_out

62

2
62
superkingdom

62
976
phylum

200643
62
class

order
62
171549

family
1853231
61

genus
61
2
283168

SRS043841\_contig\_number\_2291SRS144183\_contig\_number\_23890

59
28118

SRS013098\_contig\_number\_48537SRS013521\_contig\_number\_11721SRS014683\_contig\_number\_17501SRS017521\_contig\_number\_38716SRS017821\_contig\_number\_31080SRS018133\_contig\_number\_10643SRS018623\_contig\_number\_19350SRS018888\_contig\_number\_10189SRS019030\_contig\_number\_17250SRS019161\_contig\_number\_26156SRS019381\_contig\_number\_16625SRS019601\_contig\_number\_29197SRS019787\_contig\_number\_contig-100\_2174.74517SRS020328\_contig\_number\_39127SRS021219\_contig\_number\_10185SRS022137\_contig\_number\_20823SRS022609\_contig\_number\_contig-100\_893.256589SRS024492\_contig\_number\_2779SRS043768\_contig\_number\_17671SRS045244\_contig\_number\_3423SRS045713\_contig\_number\_4929SRS049446\_contig\_number\_12000SRS049995\_contig\_number\_30333SRS050422\_contig\_number\_contig-100\_88.197071SRS051610\_contig\_number\_contig-100\_240.69878SRS051882\_contig\_number\_11806SRS054905\_contig\_number\_20354SRS055017\_contig\_number\_33145SRS055982\_contig\_number\_18915SRS058770\_contig\_number\_28060SRS063040\_contig\_number\_32409SRS063518\_contig\_number\_39585SRS064757\_contig\_number\_20573SRS075078\_contig\_number\_contig-100\_2312.176795SRS076756\_contig\_number\_16759SRS076804\_contig\_number\_18651SRS077127\_contig\_number\_14481SRS077552\_contig\_number\_20939SRS078176\_contig\_number\_20373SRS098073\_contig\_number\_contig-100\_5.45995SRS101433\_contig\_number\_contig-100\_171.101181SRS1041118\_contig\_number\_14763SRS1041129\_contig\_number\_2743SRS1041145\_contig\_number\_24476SRS104311\_contig\_number\_15346SRS104975\_contig\_number\_12683SRS105153\_contig\_number\_34411SRS1054716\_contig\_number\_6596SRS1055076\_contig\_number\_10246SRS140492\_contig\_number\_33572SRS140513\_contig\_number\_12867SRS144297\_contig\_number\_contig-100\_3397.93806SRS144362\_contig\_number\_14293SRS144506\_contig\_number\_38141SRS146764\_contig\_number\_2493SRS147557\_contig\_number\_20858SRS148091\_contig\_number\_1844SRS148424\_contig\_number\_28016SRS893341\_contig\_number\_8266
species

1
815
family

genus
1
816

1
28116

SRS064557\_contig\_number\_107
species
